# Placental T2WI MRI-based radiomics-clinical nomogram predicts suspicious placenta accreta spectrum in patients with placenta previa

**DOI:** 10.1186/s12880-024-01328-y

**Published:** 2024-06-13

**Authors:** Hongchang Yu, Hongkun Yin, Huiling Zhang, Jibin Zhang, Yongfei Yue, Yanli Lu

**Affiliations:** 1 Department of Radiology, Affiliated Suzhou Hospital of Nanjing Medical University, Suzhou Municipal Hospital, No. 26 Daoqian Street, Gusu District, Suzhou, China; 2 Department of Obstetrics and Gynecology, Affiliated Suzhou Hospital of Nanjing Medical University, Suzhou Municipal Hospital, No. 26 Daoqian Street, Gusu District, Suzhou, China; 3 Infervision Medical Technology Co., Ltd, Beijing, China

**Keywords:** Magnetic resonance imaging, Placenta accreta spectrum, Placenta previa, Radiomics, Nomogram

## Abstract

**Background:**

The incidence of placenta accreta spectrum (PAS) increases in women with placenta previa (PP). Many radiologists sometimes cannot completely and accurately diagnose PAS through the simple visual feature analysis of images, which can affect later treatment decisions. The study is to develop a T2WI MRI-based radiomics-clinical nomogram and evaluate its performance for non-invasive prediction of suspicious PAS in patients with PP.

**Methods:**

The preoperative MR images and related clinical data of 371 patients with PP were retrospectively collected from our hospital, and the intraoperative examination results were used as the reference standard of the PAS. Radiomics features were extracted from sagittal T2WI MR images and further selected by LASSO regression analysis. The radiomics score (Radscore) was calculated with logistic regression (LR) classifier. A nomogram integrating Radscore and selected clinical factors was also developed. The model performance was assessed with respect to discrimination, calibration and clinical usefulness.

**Results:**

A total of 6 radiomics features and 1 clinical factor were selected for model construction. The Radscore was significantly associated with suspicious PAS in both the training (*p* < 0.001) and validation (*p* < 0.001) datasets. The AUC of the nomogram was also higher than that of the Radscore in the training dataset (0.891 vs. 0.803, *p* < 0.001) and validation dataset (0.897 vs. 0.780, *p* < 0.001), respectively. The calibration was good, and the decision curve analysis demonstrated the nomogram had higher net benefit than the Radscore.

**Conclusions:**

The T2WI MRI-based radiomics-clinical nomogram showed favorable diagnostic performance for predicting PAS in patients with PP, which could potentially facilitate the obstetricians for making clinical decisions.

**Supplementary Information:**

The online version contains supplementary material available at 10.1186/s12880-024-01328-y.

## Introduction

Placenta accreta spectrum (PAS) is a condition characterized by the invasion of placental villi into part or whole of the myometrium including placenta accreta, placenta increta, and placenta percreta [1, 2]. This is a serious obstetrics complication that can result in maternal hemorrhage, shock, uterine perforation, secondary infection, and even death. Increasing with the number of cesarean deliveries, prior cesarean delivery accompanied with placenta previa (PP) has become the highest risk factors for PAS [3–5]. Other factors, such as History of previous uterine curettage, and conception through artificial reproductive technologies (ART), multiparity, induced abortion, puerperium infection, history of pelvic radiotherapy, and advanced age are also considered to be risk factors for PAS [6, 7].

The incidence of PAS increases in women with previous cesarean Sect. [8]. Depending to the severity of PAS, affected women may have no choice but to accept cesarean hysterectomy. Therefore, the early diagnosis of PAS is essential for obstetricians to determine appropriate surgical procedures and reduce mortality of childbirth.

Prenatal ultrasound screening for PAS is critical and necessary, but accurate diagnosis can be challenging for cases involving posterior placenta and those with unclear or controversial results. Magnetic resonance imaging (MRI) can be an important complementary problem-solving tool with the advantage of multi-directional, multi-sequence imaging and later re-evaluations [9, 10]. Due to the especial imaging effect that it can offer better assessment for the location of the placenta and adjacent organ involvement, there is good prospect for MRI in the area of PAS diagnosis [9].

Radiologists should have a solid theoretical knowledge to analyze and diagnose the morphology and signal characteristics of placenta. Placental heterogeneity and irregular placental dark T2 bands area are the important MRI visual characterization that help diagnose PAS, but these depended to radiologists’ subjective assessment [11, 12]. However, it is noteworthy that one significant factor that affects diagnostic performance is observer experience [13]. Experienced radiologists have higher sensitivity and specificity in diagnosing PAS compared to junior radiologists (sensitivity 90.9% vs. 81.8%, specificity 75% vs. 61.8%) [11, 14]. Therefore, the simple visual feature analysis of images cannot completely and accurately reflect the state of PAS.

Radiomics is an emerging field in the application of MR medical imaging and becomes a research hotspot. It uses automated algorithms to extract image features for a series of qualitative and quantitative analysis to obtain information about diagnosis, prognosis and features of various diseases. Radiomics research is a combination of multiple disciplines and technologies [15–17]. It does not require invasive examination to extract tissue features and solves the problem of accurate evaluation of heterogeneity.

Thus, the aim of this study is to determine the value of MRI radiomics for predicting PAS using machine learning tools, and to improve diagnostic and predictive accuracy for further providing recommendations for clinical decision-making.

## Methods

### Patients

**Fig. 1:**
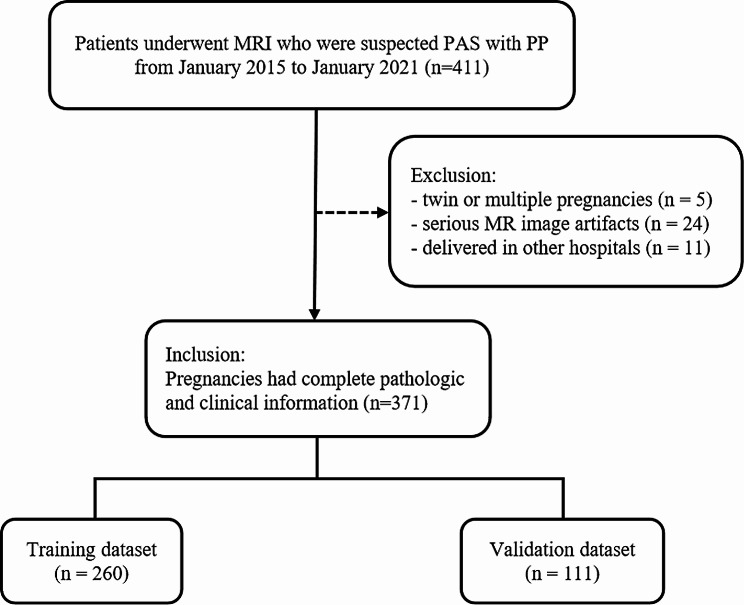
Patient enrollment and study design

Our retrospective study was approved by the Institutional Review Board (IRB) of The Affiliated Suzhou Hospital of Nanjing Medical University, Suzhou Municipal Hospital. All magnetic resonance images were collected from the picture archiving and communication system (PACS) in our hospital. A total of 411 consecutive pregnant women with PP who underwent placental MRI examinations because of suspected PAS from January 2015 to January 2021 were initially recruited. All the pregnant women had PAS proved by pathologic examination (including placenta accreta, increta, and percreta, as previously described [1]) after a placenta or cesarean hysterectomy specimen. The inclusion criteria were as follows: (1) pregnancies that underwent MRI before cesarean operation; (2) all pregnant women had complete pathologic and clinical information. In all, 40 patients were excluded for the exclusion criteria: (1) twin or multiple pregnancies (*n* = 5); (2) serious MR image artifacts affecting observation (*n* = 24); (3) delivered in other hospitals (*n* = 11). Finally, 371 patients were included in this study. The enrolled patients were randomly split into the training dataset (*n* = 260) and the validation dataset (*n* = 111) following the ratio of 7:3. The flowchart of patient enrollment was presented in Fig. 1.

### Obtainment and selection of the clinical variables

A total of 14 clinical characteristics of the enrolled pregnant women were collected from the EMR system and RIS system, including maternal age, gravidity, cesarean delivery, abortion, parturition, hypertension, pregnancy with hypothyroidism, diabetes, prenatal and postpartum vaginal bleeding, gestational age at MRI and delivery, uterine fibroids, Apgar scores, placental position and presence of implantation during and after operation.

Univariate and multivariate regression analysis were used for the selection of the clinical variables. The clinical factors which were significantly associated with PAS in the univariate analysis were chose for multivariate analysis, and only the clinical factors with a *p*-value less than 0.05 in multivariate regression analysis were used for model development.

### Acquisition of T2WI MR images

All placental magnetic resonance examinations were performed on a 1.5 T (Achieva; Philips Medical Systems) or 3.0 T (Skyra; Siemens Medical Systems) MRI. 1.5 T MR images were obtained using T2-weighted images with BTFE sequence (TR 3.3 ms, TE 1.67 ms, 240 × 219 matrix over a field of view of 350 (FH) × 321 (AP) × 159 (RL) mm, 7 mm slice thickness). 3.0 T MR images were collected through T2-weighted half-fourier acquisition single shot turbo spin echo (HASTE) sequence (TR 700 ms, TE 87 ms, 432 × 432 matrix over a field of view of 380 × 380 mm, 5 mm slice thickness) for full placenta coverage in the axial, sagittal and coronal planes.

### Region of interest (ROI) annotation

**Fig. 2:**
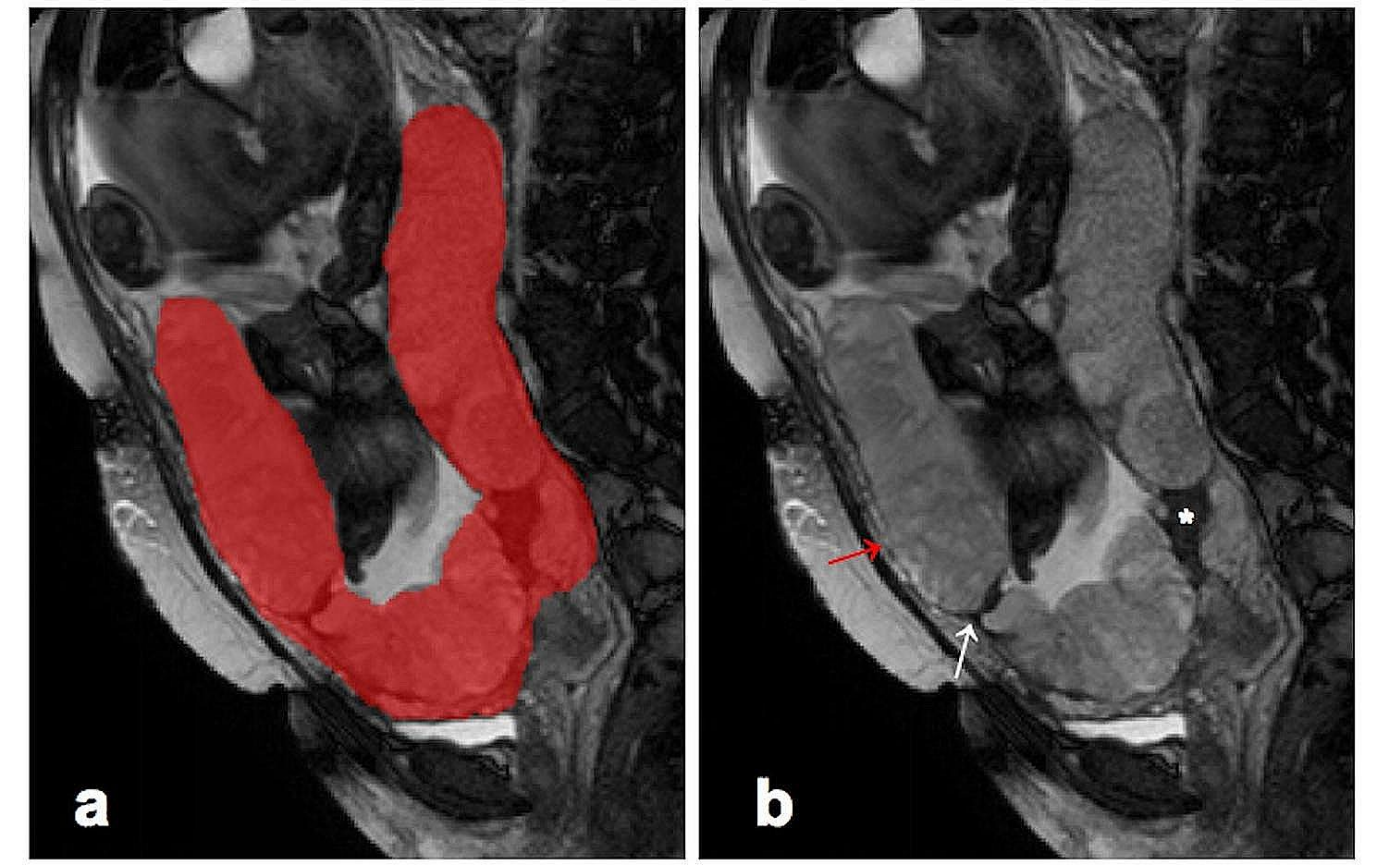
Example of ROI delineation (**a**) and the most relevant MRI signs (**b**) on the sagittal T2-weighted images in a patient with placenta accreta spectrum (PAS). Placental-myometrial interface interruption (red arrow), abnormal placental vascularity (white arrow), and placental dark T2 bands area (asterisk)

All images were retrieved from PACS and labeled on a local workstation. The sagittal T2WI was chosen because it was the best sequence to observe the placenta, and the placental region of interest (ROI) was manually delineated and segmented by a radiologist with 9 years of experience in pelvic MRI with the ITK-SNAP software. The ROIs were further reviewed by an expert with more than 10 years of experience in pelvic. Figure 2a showed the ROI was positioned including the placenta with underneath myometrium. This segmentation strategy has also shown in previous studies that seems to be the best method when using 2D segmentation [18]. Examples of placental-myometrial interface interruption, placental dark T2 bands area, abnormal placental vascularity detected on MRI were illustrated in Fig. 2b.

### Extraction and selection of radiomics features

The MR images were firstly processed with the N4 bias field correction algorithm to correct the low frequency intensity non-uniformity. Then, the radiomics features were automated extracted by using an open-sourced package named PyRadiomics (version 2.2.0). Multiple filters including Exponential, Logarithm, Log-sigma, Square, Squareroot and Wavelet were applied to highlight particular image properties. Finally, a total of 1454 radnomics features were extracted from each ROI, including 14 shape features, 288 first-order histogram statistics features and 1152 s-order texture statistics features. Subsequently, forty patients were randomly selected and re-labeled by the same radiologist after 4 weeks and the intraclass correlation coefficient (ICC) was calculated for evaluating the inter-observer reliability. Only the stable radiomics features with intra-observer ICC over 0.75 were used for further analysis.

In order to reduce computation complexity and prevent overfitting, the least absolute shrinkage and selection operator (LASSO) was applied for feature selection. The key radiomics features that were most closely associated with the PAS occurrence were selected with penalty parameter tuning conducted by ten-fold cross-validation via the one-standard-error criteria.

### Model development and performance evaluation

The z-score standardization method was applied for the normalization of selected radiomics features before model construction. The radiomics score (Radscore) was assessed for each patient via the combination of selected radiomics features and their corresponding weights. In addition, a nomogram was also constructed by integrating the Radscore the selected clinical factors.

The discriminative efficacy of the Radscore and the nomogram were evaluated and compared by the receiver operating characteristics (ROC) analysis with respect to the area under the curve (AUC). In addition, the detailed accuracy (ACC), sensitivity (SEN), specificity (SPE), positive predictive value (PPV) and negative predictive value (NPV) were also assessed under the optimal threshold which was determined by the maximum Youden index.

The model development and validation were performed with the InferScholar platform (version 3.5).

### Calibration and decision curve analysis

The calibration curve was plotted by using 1000 bootstrapping resamples method, and the consistency between the predicted PAS probability and actual rate was assessed by Hosmer–Lemeshow test. Decision curve analysis (DCA) was used to evaluate the clinical usefulness by estimating the net benefits for a range of threshold probabilities.

### Statistical analysis

The SPSS software (version 23.0) and MedCalc software (version 18.0) were used for statistical analyses. The differences between continuous variables were evaluated by the Mann–Whitney U test or Student’s t test, as appropriate. The Chi-Squared test was used for the comparison of dichotomous variables. Delong’s test was employed for the comparison of difference between two AUCs. The calibration curve and decision curve were plotted with R language by using the “rms” package (version 6.2) and the “rmda” package (version 1.6), respectively. A two-sided *p* < 0.05 was considered statistically significant.

## Results

### Patient characteristics

In total, 371 cases of PP were enrolled in this study. The mean age of pregnant women was 32.20 ± 4.33 years, ranges from 22 to 44. The gestational age at delivery was 27–39 w, and the gestational age underwent MRI was 26–39 w. 208 cases of them were approved have PAS by post-operating, 163 cases were simple PP. The statistical results showed that the mean amount of postpartum hemorrhage in the PAS group was approximately 1860.22 mL, and that in the non-PAS group was 457.45 mL, *p* < 0.001, indicated that PAS would significantly increase the risk of postpartum hemorrhage. Patient detailed clinical characteristics and statistical results in the training and validation datasets were shown in Table 1. No significant differences in the prevalence of PAS were found between the training and validation datasets (55.8% vs. 56.7 = 8%, *p* = 0.861). There were significant differences in cervical marginal sinus, placental dark T2 bands area, local placental bulge, low signal interruption at the placental-myometrial interface, abnormal placental vascularity and uterine serosal hypervascularity and previous cesarean deliveries between the non-PAS and PAS groups in both training and validation datasets (all *p* values < 0.05).

**Table 1:** Patient characteristics

Clinical variables	Training dataset (*n* = 260)				Validation dataset (*n* = 111)		
	Normal	PAS	*p-value*		Normal	PAS	*p-value*
Maternal age, years (mean ± SD)	31.7 ± 4.4	33.0 ± 4.1	*0.025*		31.3 ± 4.4	32.0 ± 4.3	*0.449*
MRI placenta previa type	85/30	120/25	*0.083*		42/6	55/8	*0.975*
Placental hyperplasia	115/0	125/20	*< 0.001*		46/2	54/9	*0.077*
Cervical marginal sinus	100/15	89/56	*< 0.001*		45/3	47/16	*0.008*
Placental dark T2 bands area	81/34	68/77	*< 0.001*		32/16	29/34	*0.030*
Local placental bulge	109/6	103/42	*< 0.001*		48/0	45/18	*< 0.001*
Placental-myometrial interface interruption	96/19	22/123	*< 0.001*		45/3	11/52	*< 0.001*
Abnormal placental vascularity	99/16	93/52	*< 0.001*		45/3	47/16	*0.008*
Uterine serosal hypervascularity	113/2	123/22	*< 0.001*		48/0	57/6	*0.028*
Gravidity (mean ± SD)	2.71 ± 1.24	3.48 ± 1.46	*< 0.001*		2.94 ± 1.45	3.40 ± 1.61	*0.126*
Previous caesarian deliveries (mean ± SD)	0.42 ± 0.57	0.79 ± 0.60	*< 0.001*		0.46 ± 0.54	0.89 ± 0.78	*0.002*
Abortion history (mean ± SD)	0.97 ± 0.89	1.53 ± 1.29	*< 0.001*		1.21 ± 1.14	1.38 ± 1.28	*0.465*
Parturition (mean ± SD)	0.32 ± 0.58	0.16 ± 0.38	*0.007*		0.29 ± 0.54	0.13 ± 0.33	*0.052*
Gestational hypertension	112/3	131/14	*0.022*		45/3	55/8	*0.260*
Pregnancy with hypothyroidism	111/4	140/5	*0.990*		45/3	59/4	*0.983*
Gestational diabetes	95/20	119/26	*0.910*		41/7	52/11	*0.684*
Vaginal bleeding during pregnancy	57/43/15	86/45/14	*0.282*		22/16/10	37/17/9	*0.386*
Gestational age at delivery (week, mean ± SD)	36.2 ± 1.4	35.9 ± 1.5	*0.058*		36.4 ± 1.2	36.1 ± 1.2	*0.165*
Fetal gender	53/62	69/76	*0.810*		22/26	31/32	*0.725*
Fetal birth weight (gram, mean ± SD)	2807.0 ± 78.9	2739.2 ± 59.8	*0.206*		2804.2 ± 92.9	2787.3 ± 00.3	*0.827*

### Selection of the clinical variables

The univariate regression analysis showed that the parturition, cervical marginal sinus, local placental bulge, abortion, maternal age, previous caesarian deliveries, gravidity, gestational hypertension, placental-myometrial interface interruption, placental dark T2 bands area, abnormal placental vascularity and uterine serosal hypervascularity were associated with the outcome event (PAS) and used for further analysis. After multivariate regression analysis, only placenta-myometrial interface interruption showed a *p*-value less than 0.05 and were selected for model development (Table 2).

**Table 2:** Univariate and multivariate regression analysis of the clinical factors

Variable	Univariate regression analysis				Multivariate regression analysis		
	Odds ratio	95% CI	*P*		Odds ratio	95% CI	*P*
MRI placenta previa type	0.590	0.324–1.0746	*0.085*				
Amount of antepartum bleeding	0.756	0.529–1.0818	*0.126*				
Parturition	0.490	0.284–0.8436	*0.010*		5.04E + 07		*0.999*
Gestational age at delivery	0.842	0.702–1.0093	*0.063*				
Cervical marginal sinus	4.195	2.218–7.9347	*< 0.001*		1.470	0.610–3.541	*0.391*
Local placental bulge	7.408	3.021–18.1622	*< 0.001*		1.554	0.503–4.797	*0.443*
Abortion history	1.585	1.247–2.0137	*< 0.001*		1.88E + 08		*0.998*
Maternal age, years	1.069	1.008–1.1331	*0.026*		0.989	0.900–1.087	*0.813*
Previous caesarian deliveries	2.948	1.883–4.6158	*< 0.001*		1.88E + 08		*0.998*
Gravidity	1.522	1.251–1.8512	*< 0.001*		8.56E–09		*0.998*
Gestational hypertension	3.990	1.118–14.2388	*0.033*		2.080	0.386–11.204	*0.394*
Pregnancy with hypothyroidism	0.998	0.262–3.8056	*0.998*				
Gestational diabetes	1.038	0.546–1.9727	*0.910*				
Fetal birth weight	1.000	0.999–1.000	*0.206*				
Fetal gender	0.942	0.577–1.538	*0.810*				
Placental-myometrial interface interruption	28.249	14.464–55.170	*< 0.001*		15.800	7.134–34.990	*< 0.001*
Placental dark T2 bands area	2.698	1.609–4.522	*< 0.001*		1.238	0.589–2.602	*0.574*
Abnormal placental vascularity	3.460	1.847–6.481	*< 0.001*		2.108	0.844–5.267	*0.110*
Placental hyperplasia	1.87E + 09		*0.997*				
Uterine serosal hypervascularity	10.106	2.324–43.948	*0.002*		3.972	0.729–21.648	*0.111*

### Selection of the radiomics features

**Fig. 3:**
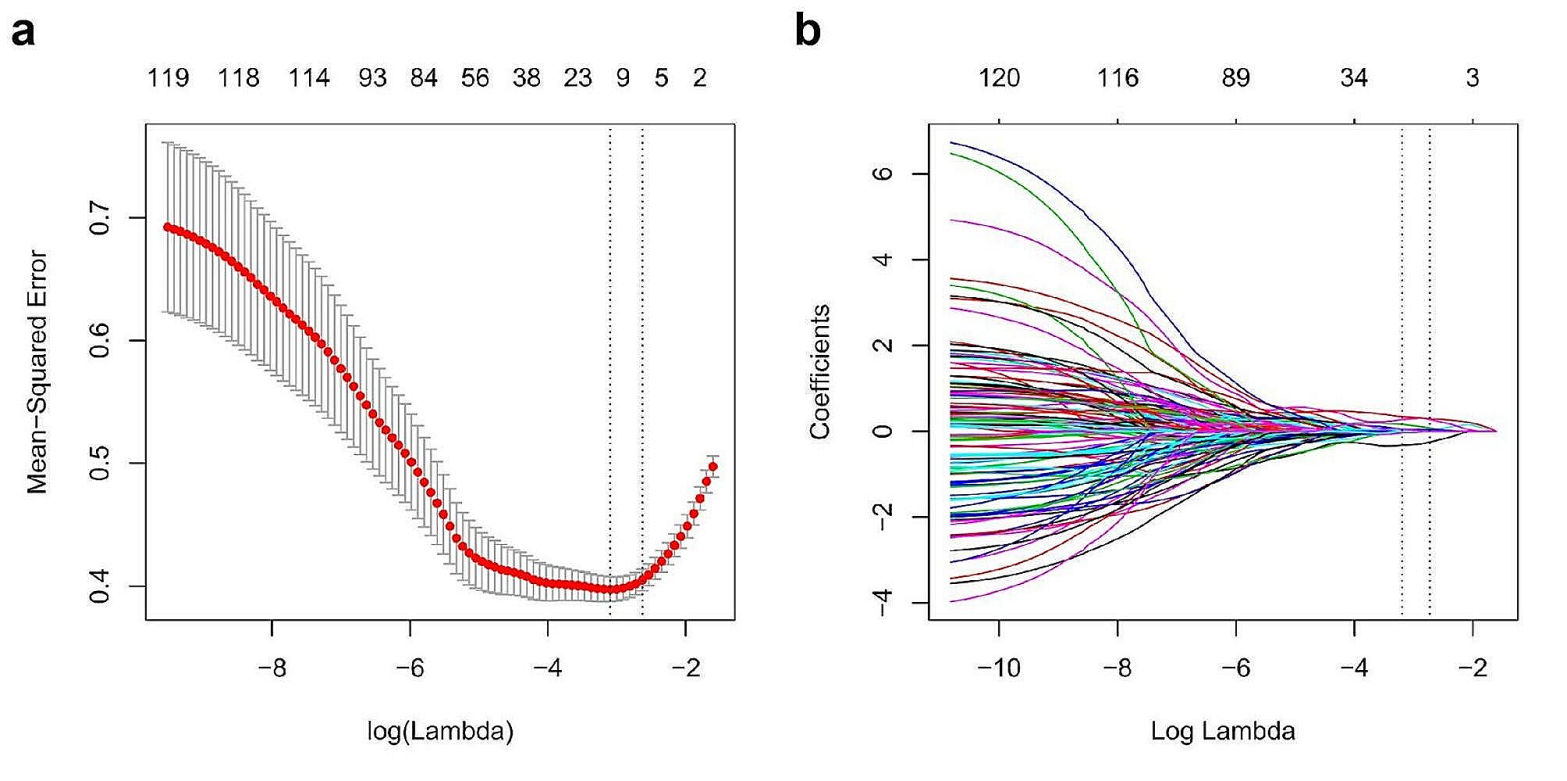
LASSO regression analysis. (**a**) Tuning parameter lambda selection using 1SE criteria via 10-fold cross-validation method. (**b**) The coefficient profile against the optimal log (lambda) sequence

**Fig. 4:**
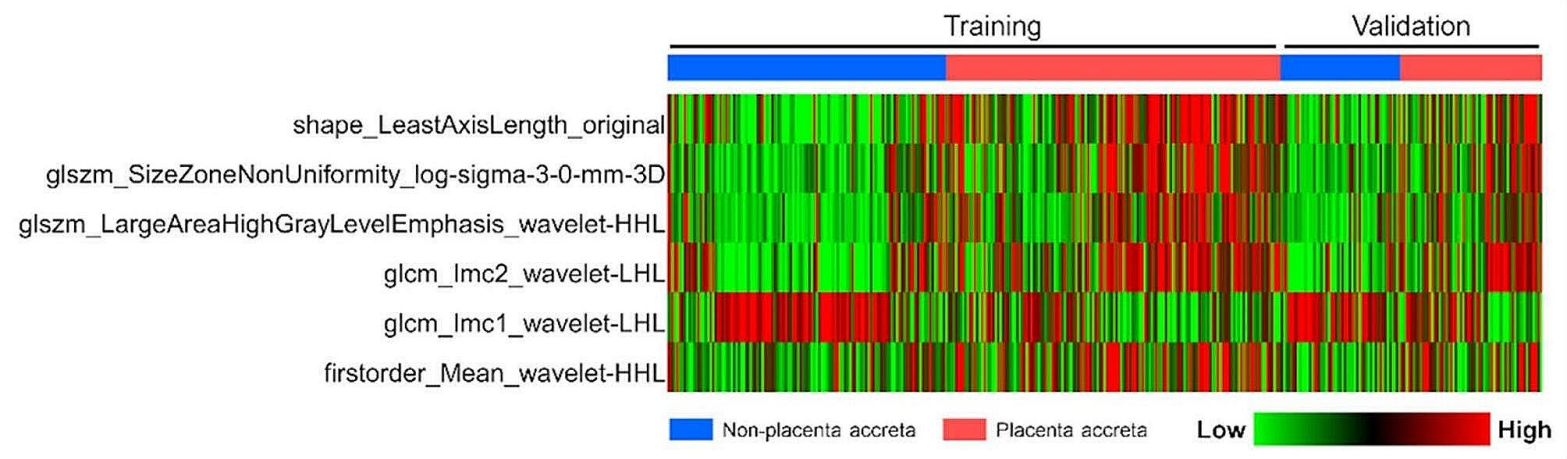
Heatmap of the six selected radiomics features in the training dataset and the validation dataset. Each row represented a radiomic feature, and each column corresponded to one patient

A total of 1073 stable radiomics features with ICCs > 0.75 were selected by LASSO regression analysis, and ultimately 6 radiomics features with non-zero coefficients at the optimal log(lambda) sequence (lambda = 0.0688) were chosen for model development (Fig. 3). The heatmap of the 6 selected radiomics features was plotted according to the normalized radiomics feature values (Fig. 4).

### Construction of the Radscore and the nomogram

The Radscore was calculated with the following fomula:

Radscore = 0.43056 + 0.34145 × firstorder_Mean_wavelet_HHL + 0.41526 × shape_LeastAxisLength_original − 0.65948 × glcm_Imc1_wavelet_LHL − 0.24023 × glcm_Imc2_wavelet_LHL + 0.11682 × glszm_LargeAreaHighGrayLevelEmphasis_wavelet_HHL + 0.72531 × glszm_SizeZoneNonUniformity_log_sigma_3_0_mm_3D.

**Fig. 5:**
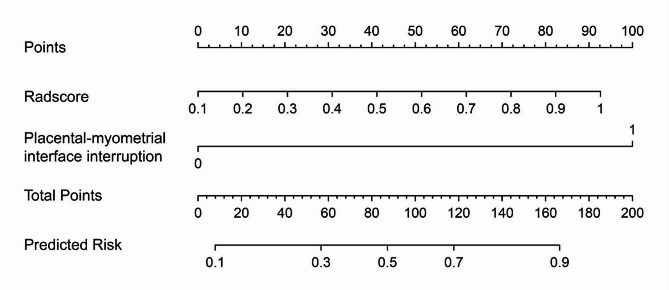
The nomogram integrating the Radscore and the selected clinical factor

In addition, an easy-to-use nomogram was also constructed by incorporating the Radscore and the placental-myometrial interface interruption in the training dataset (Fig. 5).

### Evaluation of model performance

**Fig. 6:**
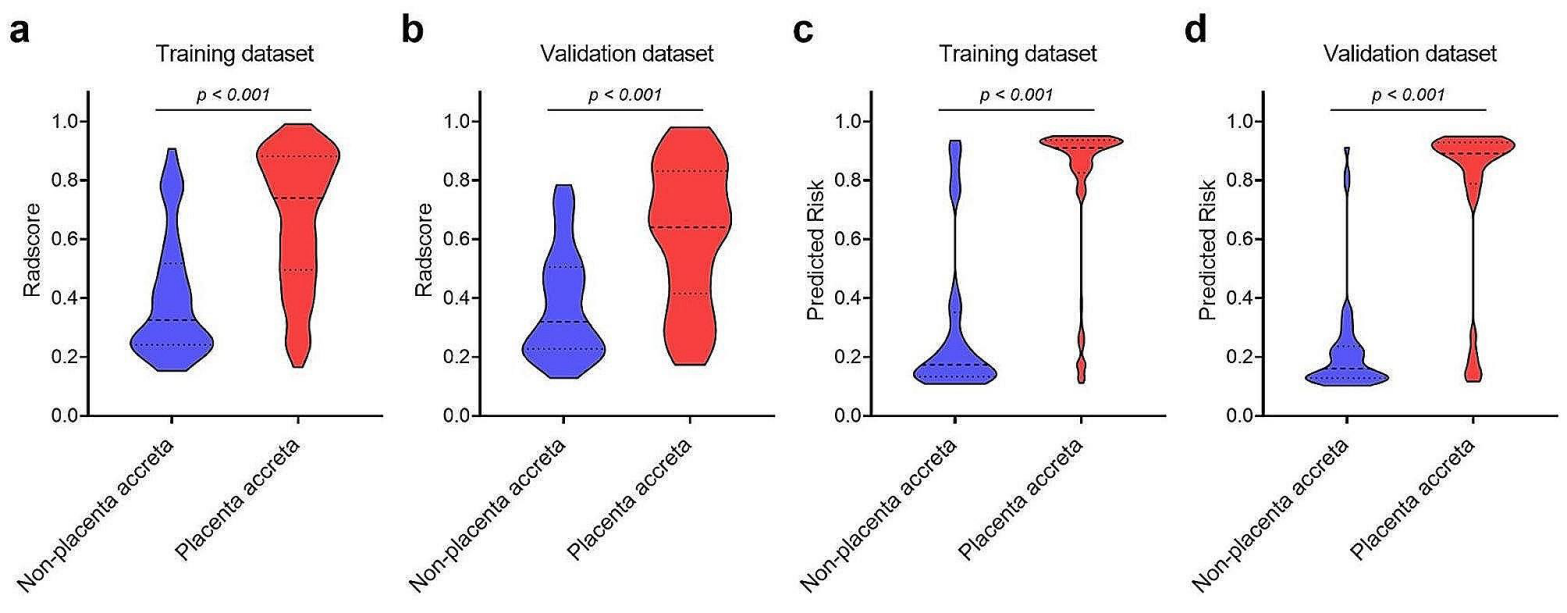
Comparison of Radscore and the predicted risk calculated by the nomogram between the PAS group and non-PAS group in the training and validation datasets. (**a**) Radscore comparison in the training dataset. (**b**) Radscore comparison in the validation dataset. (**c**) Nomogram predicted risk comparison in the training dataset. (**d**) Nomogram predicted risk comparison in the validation dataset

**Fig. 7:**
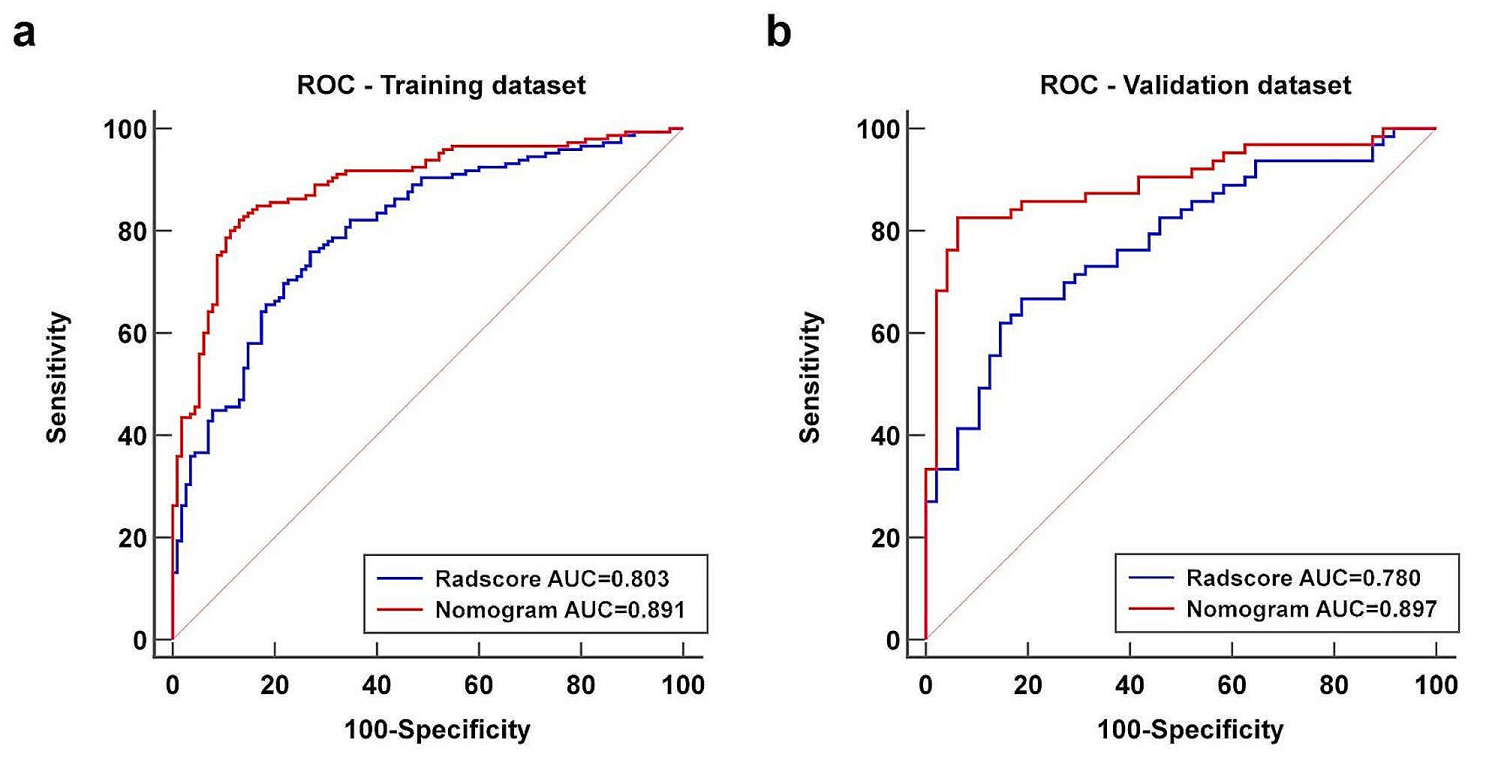
ROC analysis of the Radscore and the nomogram in the training dataset (**a**) and the validation dataset (**b**)

Both the MRI-based Radscore and the nomogram were significantly associated with the PAS in both training and validation datasets (Fig. 6). The Radscore showed favorable performance and the AUCs were 0.803 (95% CI, 0.750–0.850) in the training dataset and 0.780 (95% CI, 0.691–0.853) in the validation dataset, respectively. The discrimination capability of the nomogram was significantly higher than that of the Radscore, with AUCs yielding 0.891 (95% CI, 0.847–0.926) in the training dataset (*p* < 0.001) and 0.897 (95% CI, 0.825–0.947) in the validation dataset (*p* < 0.001), respectively (Fig. 7). The detailed ACC, SEN, SPN, PPV and NPV of the Radscore and the nomogram were summarized in Table 3.

**Table 3:** Performance of the predictive models in the training and validation datasets

Dataset	Model	AUC (95% CI)	*p*-value	ACC	SEN	SPE	PPV	NPV
Training	Radscore	0.803 (0.750–0.850)	*< 0.001*	74.6%	75.9%	73.0%	78.0%	70.6%
	Nomogram	0.891 (0.847–0.926)		84.2%	82.1%	87.0%	88.8%	79.4%
Validation	Radscore	0.780 (0.691–0.853)	*< 0.001*	73.0%	66.7%	81.3%	82.4%	65.0%
	Nomogram	0.897 (0.825–0.947)		87.4%	82.5%	93.8%	94.5%	80.4%

### Clinical utility analysis

**Fig. 8:**
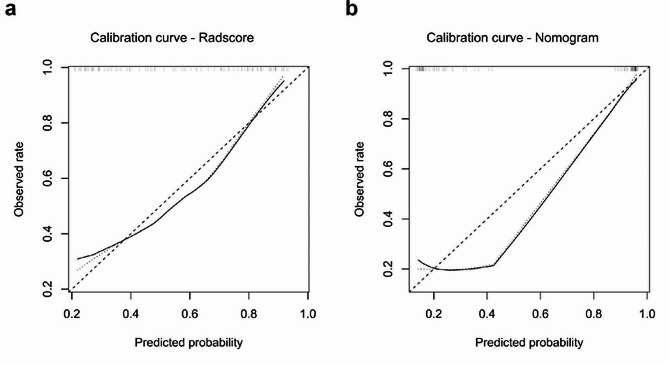
Calibration analysis of the Radscore (**a**) and the nomogram (**b**) in the validation dataset

**Fig. 9:**
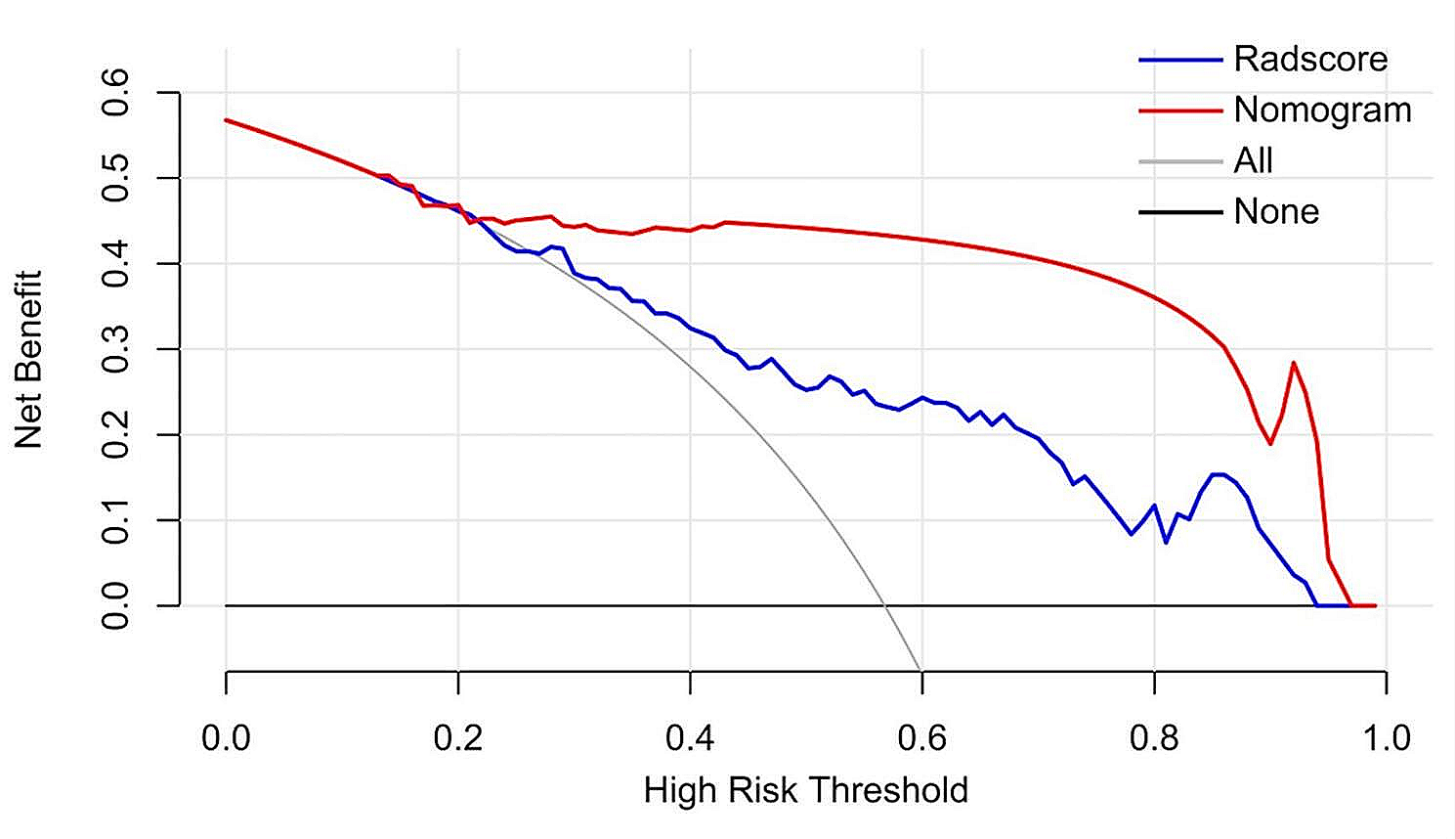
Decision curve analysis in the validation dataset

Both the Radscore and the nomogram exhibited good agreement between the predicted PAS probability and the actual observed rate in the validation dataset (Fig. 8). The non-significant statistic of the Hosmer–Lemeshow test for the Radscore (*p* = 0.475) and the nomogram (*p* = 0.846) suggested no significant deviation from an ideal fitting. The decision curve analysis in the validation dataset was presented in Fig. 9. The net benefit of the nomogram was higher than the Radscore across almost entire range of threshold probabilities, indicating that the nomogram had better clinical utility.

## Discussion

PP is a serious complication of pregnancy that refers to the placenta attached at a lower position, partially or totally covering the internal orifice of the cervix. This condition is mostly caused by endometrial damage and multiple uterine cavity operations [19]. PP is the main cause of severe hemorrhage and hysterectomy in pregnant women.

PAS refers to abnormal placental attachment caused by multiple intrauterine operations, PP and other factors, which invades or penetrates the myometrium, and may even invade adjacent organs, causing the patient to have symptoms such as uterine rupture and abdominal bleeding. The prevalence of PAS in all pregnancies is about 0.4% [20]. PAS is a significant cause for postpartum hemorrhage [5], and is considered a serious obstetric emergency that can lead to maternal death [21–23].

King et al. demonstrated that women with a prior cesarean delivery were seven times more likely to have persistent placenta previa (PPP) than without [24]. Ogawa et al. found that the history of cesarean section was the strongest risk factor for PAS among women with PP, and that PAS patients with PP had a higher risk of blood transfusion and hysterectomy [5]. However, with the opening of China’s three-child policy and the high rate of primary cesarean section, obstetricians are facing a serious clinical problem: “placenta previa with scar uterus”, that is, dangerous PP [25]. The dangerous PP greatly increases the probability of PAS.

Therefore, the study of PP and PAS is of significant clinical importance due to its association with fetal and maternal mortality. All the subjects included in our study are pregnant women with PP making them more easily to develop PAS, which require more strict analysis of imaging features, and are also more in line with the urgent difficulties in imaging diagnosis.

In patients with PP, the routine assessment of clinical factors and ultrasound is useful for the preliminary judgment of the high-risk population of PAS. Romeo et al. [26] showed that either clinical factors or ultrasound and MRI signs is helpful in predicting PAS. However, the accuracy of MRI is significantly higher than that of ultrasound and clinical factors, which can reduce the incidence of false positive and false negative cases in ultrasound, and the accuracy of MRI alone in predicting PAS is significantly higher than that combining clinical factors and ultrasound. Our results showed that at univariate analysis, previous caesarian deliveries, parturition, abortion, maternal age, gravidity, gestational hypertension, were correlated with PAS. In terms of MR imaging signs [12, 27, 28], placental-myometrial interface interruption, placental dark T2 bands area, abnormal placental vascularity and uterine serosal hypervascularity, cervical marginal sinus, local placental bulge were significantly correlated with PAS. At multivariate analysis, placental-myometrial interface interruption was independently associated with PAS, consistent with previous study [26, 29]. Alamo et al. specifically reported the high diagnostic sensitivity (91%) of this sign [14]. Lax et al. described that placental heterogeneity and placental dark T2 bands area are important and visible MRI marker of PAS [12]. The placental dark T2 bands area is usually considered to be the result of fibrin deposition or placental infarction [3, 27]. Local placental bulge indicates the role of extensive growth of placental villus [3, 30]. These texture features are consistent with those identified by our research. However, these textural features are difficult to be quantified by radiologists [31–33]. Radiomics has the potential feasibility to achieve quantization [16, 34]. Integrating multiple features which are associated with PAS can greatly improve diagnostic efficiency [3].

In recent years, some studies have reported the value of MRI based radiomics model in predicting PAS. Sun [11] and Romeo [35] et al. constructed a model to predict PAS using MRI textural features and automated machine learning. Compared to their studies, as a diagnosis and treatment center for high-risk pregnant women, our research features a larger sample size and lower lost follow-up rate. Moreover, we compared the predictive value between Radscore and the nomogram, and proved that the importance of radiomics combined with clinical variables.

Our study identified an automated machine learning algorithm capable of predicting the PAS in pregnant women with high-risk factors or those clinically suspected of having the condition. The results showed that the MRI-based Radscore and the nomogram were both significantly associated with the PAS in training and validation datasets. More than this, the discrimination capability of the nomogram was significantly higher than that of the Radscore, with the SEN (82.1% vs. 75.9%), SPE (87.0% vs. 73.0%) in the training and the SEN (82.5% vs. 66.7%), SPE (93.8% vs. 81.3%) in the validation. This suggested that the MRI-based radiomics-clinical nomogram performed higher accuracy in MRI pre-delivery prediction.

In clinical work, it can help obstetricians to triage high-risk parturients, and then make necessary preparations for reducing the incidence of massive hemorrhage and hysterectomy caused by placenta implantation during spontaneous labor. It will be feasible and promising to integrate the models into image workstation to help imaging doctors make more accurate diagnosis quickly.

There are also several study limitations. We screened 6 texture features and 1 clinical feature for model development. However, it is possible that there may be other valuable predictors were not considered in this study. Besides, as a single center research, this study may face challenges in its popularization and application. In addition, it is inevitable that there may be heterogeneity bias due to MRI images were got from different MR scanners. In order to avoid this situation, all these images were normalized before feature extraction.

In addition, Stanzione et al. [36] identified and appraised the methodological quality of radiomics studies of recently published articles focused PAS disorders applications using the Radiomics Quality Score (RQS) [37, 38]. The result was that they had a median RQS of 8 and a maximum and minimum of 17/36 and − 6/36, respectively [36]. It is pointed out that the main problems of current methods are lack of feature stability and poor data openness. Accordingly, we scored our study methodology with an RQS of 19 (supporting information). Although our study has certain limitations in data openness, it is undeniable that it has diagnostic predictive value, and perhaps more powerful research methods are needed in the future to promote progress in this field and possible clinical translation.

## Conclusions

This study introduces a new artificial intelligence diagnostic approach for clinical diagnosis of PAS. The results demonstrate that the combination of omics model and clinical factors improved the diagnostic performance. It is helpful for radiologists to make more accurate diagnosis by systematically integrating imaging and clinical information, and further provides evidence for obstetricians to make clinical decisions when receiving pregnant women with suspected PAS.

### Electronic supplementary material

Below is the link to the electronic supplementary material.

Supplementary Material 1

## Data Availability

The data that support the findings of this study are available from the corresponding author upon reasonable request.
