# Supplementary material for: Placental T2WI MRI-based radiomics-clinical nomogram predicts suspicious placenta accreta spectrum in patients with placenta previa
Source: BMC Med Imaging. 2024 Jun 13;24:146. doi: 10.1186/s12880-024-01328-y (PMC11177524; doi:10.1186/s12880-024-01328-y)
Supplement: Supplementary file 1 — Supplementary Material 1 [file 12880_2024_1328_MOESM1_ESM.pdf]

Image protocol quality - well-documented image protocols (for example, contrast, slice thickness, energy, etc.) and/or usage of public image protocols allow reproducibility/replicability

☑ protocols well documented

☐ public protocol used

☐ none

Multiple segmentations - possible actions are: segmentation by different physicians/algorithms/software, perturbing segmentations by (random) noise, segmentation at different breathing cycles. Analyse feature robustness to segmentation variabilities

◉ yes

○ no

Phantom study on all scanners - detect inter-scanner differences and vendor-dependent features. Analyse feature robustness to these sources of variability

○ yes

◉ no

Imaging at multiple time points - collect images of individuals at additional time points. Analyse feature robustness to temporal variabilities (for example, organ movement, organ expansion/shrinkage)

◉ yes

○ no

Feature reduction or adjustment for multiple testing - decreases the risk of overfitting. Overfitting is inevitable if the number of features exceeds the number of samples. Consider feature robustness when selecting features

◉ Either measure is implemented

○ Neither measure is implemented

Multivariable analysis with non radiomics features (for example, EGFR mutation) - is expected to provide a more holistic model. Permits correlating/inferencing between radiomics and non radiomics features

◉ yes

○ no

Detect and discuss biological correlates - demonstration of phenotypic differences (possibly associated with underlying gene–protein expression patterns) deepens understanding of radiomics and biology

○ yes

◉ no

Cut-off analyses - determine risk groups by either the median, a previously published cut-off or report a continuous risk variable. Reduces the risk of reporting overly optimistic results

○ yes

◉ no

Discrimination statistics - report discrimination statistics (for example, C-statistic, ROC curve, AUC) and their statistical significance (for example, p-values, confidence intervals). One can also apply resampling method (for example, bootstrapping, cross-validation)

☑ a discrimination statistic and its statistical significance are reported

☑ a resampling method technique is also applied

☐ none

Calibration statistics - report calibration statistics (for example, Calibration-in-the-large/slope, calibration plots) and their statistical significance (for example, P-values, confidence intervals). One can also apply resampling method (for example, bootstrapping, cross-validation)

☑ a calibration statistic and its statistical significance are reported

☑ a resampling method technique is applied

☐ none

Prospective study registered in a trial database - provides the highest level of evidence supporting the clinical validity and usefulness of the radiomics biomarker

○ yes

◉ no

Validation - the validation is performed without retraining and without adaptation of the cut-off value, provides crucial information with regard to credible clinical performance

☐ No validation

☑ validation is based on a dataset from the same institute

☐ validation is based on a dataset from another institute

☐ validation is based on two datasets from two distinct institutes

☐ the study validates a previously published signature

☐ validation is based on three or more datasets from distinct institutes

Comparison to 'gold standard' - assess the extent to which the model agrees with/is superior to the current 'gold standard' method (for example, TNM-staging for survival prediction). This comparison shows the added value of radiomics

◉ yes

○ no

Potential clinical utility - report on the current and potential application of the model in a clinical setting (for example, decision curve analysis).

◉ yes

○ no

Cost-effectiveness analysis - report on the cost-effectiveness of the clinical application (for example, QALYs generated)

○ yes

◉ no

Open science and data - make code and data publicly available. Open science facilitates knowledge transfer and reproducibility of the study

☑ scans are open source

☑ region of interest segmentations are open source

☐ the code is open sourced

☐ radiomics features are calculated on a set of representative ROIs and the calculated features and representative ROIs are open source

Total score

19
(52.78%)
